# Efficacy of N-acetylcysteine in preventing atrial fibrillation after cardiac surgery: a meta-analysis of published randomized controlled trials

**DOI:** 10.1186/1471-2261-14-52

**Published:** 2014-04-16

**Authors:** Xue-Hui Liu, Chun-Yan Xu, Guang-Hui Fan

**Affiliations:** 1 Department of Cardiology, Wuhan General Hospital of Guangzhou Military Command, 627 Wuluo Road, Wuhan, China; 2 Department of Endocrinology, Wuhan General Hospital of Guangzhou Military Command, 627 Wuluo Road, Wuhan, China; 3 Hubei University of Chinese Medicine, 1 Tanhualin Road, Wuhan, China

**Keywords:** N-acetylcysteine, Postoperative atrial fibrillation, Cardiac surgery, Meta-analysis

## Abstract

**Background:**

Atrial fibrillation is a common complication after cardiac surgery. The aim of this study is to evaluate whether N-acetylcysteine (NAC) could prevent postoperative atrial fibrillation (POAF).

**Methods:**

PubMed, Embase and Cochrane Center Register of Controlled Trials were searched from the date of their inception to 1 July 2013 for relevant randomized controlled trials (RCTs), in which NAC was compared with controls for adult patients undergoing cardiac surgery. Outcome measures comprised the incidence of POAF, all-cause mortality, length of intensive care unit (ICU) stay, hospital length of stay, and the incidence of cerebrovascular events. The meta-analysis was performed with the fixed-effect model or random-effect model according to the heterogeneity.

**Results:**

We retrieved ten studies enrolling a total of 1026 patients. Prophylactic NAC reduced the incidence of POAF (OR 0.56; 95% CI 0.40 to 0.77; *P* < 0.001) and all-cause mortality (OR 0.40; 95% CI 0.17 to 0.93; *P* = 0.03) compared with controls, but failed to reduce the stay in ICU and overall stay in hospital. No difference in the incidence of cerebrovascular events was observed.

**Conclusions:**

Prophylactic use of NAC could reduce the incidence of POAF and all-cause mortality in adult patients undergoing cardiac surgery. However, larger RCTs evaluating these and other postoperative complication endpoints are needed.

## Background

Atrial fibrillation (AF) is a common complication that occurs after cardiac surgery. The incidence of postoperative atrial fibrillation (POAF) ranges from 10% to 65% depending on the type of surgery, perioperative characteristics, methods of monitoring and the definition of AF [1,2]. Previous studies [2-7] indicated that AF is associated with prolonged length of hospital stay, risk of stroke, and mortality, thus extensive research has been conducted to explore the mechanism of POAF and identify the effective method for preventing POAF. Beta-blockers and amiodarone are used widely to minimize the risk of POAF and recommended by current guidelines [8]. However, their use requires caution because of potential drug-related side effects. Clinical studies have demonstrated higher inflammatory cytokines level and oxidative damage in patients who developed POAF versus those who did not after undergoing cardiac surgery, suggesting that oxidative stress and inflammatory reaction contribute to POAF [9-11].

N-acetylcysteine (NAC) is an antioxidant and anti-inflammatory agent, and could reduce cellular oxidative damage and systematic inflammation during cardiac surgery [12,13]. Previous meta-analyses showed that the NAC supplementation effectively reduced the incidence of POAF [14,15]. However, of all the included studies, only one [12] treated POAF as primary endpoint and showed a positive result. Recently, a large trial demonstrated that there was no statistical difference in the incidence of POAF between the NAC and placebo groups [16]. Thus, we conducted an updated meta-analysis to further evaluate the efficacy of NAC on the prevention of POAF in adult patients undergoing cardiac surgery. Besides, we also assessed whether NAC could reduce hospital length of stay, intensive care unit (ICU) stay, all-cause mortality, and cerebrovascular events.

## Methods

### Literature search

A comprehensive search was performed to identify all published randomized controlled trials (RCTs) of NAC versus control during cardiac surgery in any language. PubMed, Embase and Cochrane Central Register of Controlled Trials databases were searched from the date of their inception to 1 July 2013. Searched terms included N-acetylcysteine, acetylcysteine, acetadote, mucomyst, heart surgery, cardiac surgery, cardiothoracic surgery, cardiopulmonary bypass, CPB, coronary artery bypass graft, CABG, valve surgery, valvular surgery and atrial fibrillation.

### Inclusion and exclusion criteria

Only RCTs reporting the use of NAC in the prevention of POAF were included in the meta-analysis (including those reporting the concomitant use of other anti-arrhythmic agents). Exclusion criteria included: (1) duplicated data; (2) laboratory study; (3) abstract, review or letter to editor; and (4) patient age less than 18 years. Based on these criteria, two investigators (Liu and Xu) independently selected studies for further screening by reading title and/or abstract of all identified literatures. All potential eligible studies were obtained for further assessment.

### Data extraction

Two investigators (Liu and Xu) independently extracted the following information from each article: first author’s name, year of publication, country of origin, surgery type, perioperative characteristics, NAC protocol, incidence of POAF, length of ICU and hospital stay, all-cause mortality and cerebrovascular events. The primary outcome was the incidence of POAF. The length of hospital and ICU stay, all-cause mortality, and the incidence of cerebrovascular events were considered secondary outcomes. Disagreements were resolved by consensus.

### Quality assessment

The methodological quality of the studies included in the meta-analysis was assessed using validated Jadad 5 point scale [17]. This system emphasizes on the following three parts when defining the quality of a RCT: (1) randomization; (2) blinding; and (3) description of withdrawals and dropouts. A score of one is given for each of the points described. A further point is obtained where the method of randomization and/or blinding is given and is appropriate; where it is inappropriate, a point is deducted. Studies with a score ≤ 2 were considered low quality, and studies with a score >2 were considered high quality.

### Statistical analysis

Outcomes were treated as dichotomous (when incidence was reported) or continuous (when the mean and standard deviation were reported) variables. For dichotomous variables, odds ratios (ORs) and 95% confidence intervals (CIs) were calculated. For continuous variables, the weighted mean difference (WMD) was calculated. Heterogeneity was explored using I^2^, which describes the percentage of total variation across trials due to heterogeneity rather than chance alone. When I^2^ was more than 50%, significant statistical heterogeneity was considered to be present [18]. Pooled estimates of efficacy were calculated using the fixed-effects model. If there was heterogeneity, the random-effects model was used. Sensitivity analyses were conducted to test the robustness of overall pooled effect. The presence of publication bias was evaluated by using funnel plot. A two-tailed P-value < 0.05 was considered as significant difference. All statistical analyses were performed using Review Manager version 5.2.

## Results

### Identification of eligible studies

**Figure 1:**
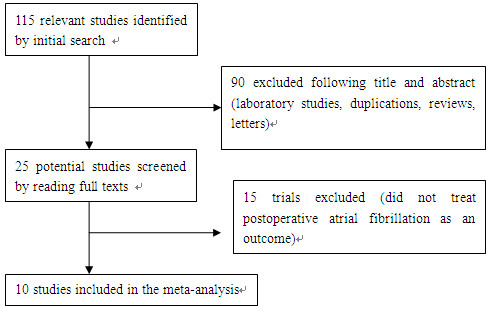
Flow diagram of trials included in the meta-analysis.

One hundred and fifteen articles were identified by the initial literature search, and 90 studies were excluded after title and abstract screening. Therefore, 25 potential relevant studies were retrieved to read the entire manuscript. Fifteen studies were further excluded because they did not treat POAF as an outcome. Consequently, 10 trials (n = 1026 patients) were included in this meta-analysis (Figure 1).

### Characteristics of eligible studies

Patients’ characteristics of the included studies are shown in Table 1. The number of patients ranged from 20 [19,20] to 240 [16]. Of the 10 RCTs, five trials were conducted in Turkey [12,19-22], two in Canada [23,24], one in Iran [16], one in Germany [25], and one in Korea [26]. With the use of Jadad 5 point score, all studies were considered high quality. Both male and female were included in all trials. Only one study [25] included some patients with a history of AF. Diabetic mellitus, hypertension, chronic heart failure, and coronary artery disease were the major concomitant diseases. Five trials [19,20,22,24,26] enrolled 228 patients undergoing CABG only, and the remaining 5 trials [12,16,21,23,25] included 798 patients undergoing valve surgery or combination valve surgery with CABG. NAC administration regimen is also presented in detail in Table 1, with 8 studies [12,19-23,25,26] using intravenous administration and 2 studies [16,24] using oral administration before cardiac surgery. The duration of NAC administration after cardiac surgery varied from 4 hours [23] to 72 hours [16]. Only one study [21] investigated the efficacy of beta-blocker (carvedilol) with NAC on the prevention of POAF.

**Table 1:** Characteristics of the included studies

**Reference**	**Jadad score**	**Surgery type**	**Number NAC/control**	**NAC protocol**	**Medical history**	**Previous medication**
Ozaydin 2008 [12]	4	CABG ± valve	58 /57	50 mg/kg iv for 1 h before surgery, then 50 mg/kg/day 48 h after operation	DM, Hypertension, CAD	BRB, ACEI, Statins, Acetylsalicylic acid
Kazemi 2013 [16]	5	CABG ± valve	120 /120	1200 mg orally 2 times per day from 48 h before and up to 72 h after heart surgery	DM,CRF,CAD, CHF, CLD, Hypertension, Hyperlipidemia	BRB,Statins, ACEI/ARB, Digoxin, Diuretic
Eren 2003 [19]	3	CABG	10 /10	100 mg/kg iv for 1 h before and 40 mg/kg/day at 24 h after CPB	CLD	Not reported
Orhan 2006 [20]	3	CABG	10 /10	50 mg/kg iv at the start of induction of anesthesia for 30 minutes	DM, CAD, Hypertension, Hyperlipidemia	Not reported
Ozaydin 2013 [21]	5	CABG ± valve	104 /104	50 mg/kg iv 1 h before and at the same does for 48 h after surgery	CHF, CLD, DM, CAD Hypertension	BRB, ACEI/ARB, Statins
Peker 2008 [22]	4	CABG	19 /21	50 mg/kg iv 1 h before surgery and 50 mg/kg/day 48 h after the operation	Not reported	Not reported
Wijeysundera 2007 [23]	5	CABG ± valve	88 /87	100 mg/kg iv over 30 min after induction of anesthesia, then 20 mg/kg/h for 4 h after CPB	CHF, CLD, DM, CVD, PVD, Hypertension	BRB, CCB, ACEI/ARB, NSAIDs
EI-Hamamsy 2007 [24]	3	CABG	50 /50	600 mg orally the day before and the morning of the operation, 150 mg/kg iv before skin incision, then 12.5 mg/kg/h over 24 h	CHF, CAD	BRB, CCB, ACEI
Haase 2007 [25]	5	CABG ± valve	30 /30	150 mg/kg iv after anesthesia induction, then 50 mg/kg iv over 4 h, then 100 mg/kg iv over 20 h	DM, CLD, CAD, PVD, Stroke, Hypertension, Hyperlipidemia, Carotid disease	Not reported
Kim 2011 [26]	4	CABG	24 /24	100 mg/kg iv bolus after anesthetic induction, then 40 mg/kg/day iv for 24 h	DM, Hypertension	BRB, CCB, ACEI, Diuretics

ACEI/ARB, angiotensin converting enzyme inhibitor/angiotensin receptor blocker; BRB, beta-receptor blocker; CAD, coronary artery disease; CCB, calcium channel blocker; CHF, chronic heart failure; CLD, chronic lung disease; CRF, chronic renal failure; SD, standard deviation; DM, diabetes mellitus; CVD, cerebrovascular disease; NSAIDs, non-steroid anti -inflammatory drugs; PVD, peripheral vascular disease.

### Incidence of POAF

The data of the included studies are shown in Table 2. The method of monitoring and the definition of POAF are presented in Table 3. Of the 10 trials, only four [12,16,21] treated POAF as their primary endpoint.

**Table 2:** Outcomes of included studies in the meta-analysis

**Reference**	**POAF**		**Duration of ICU (hours)**		**Hospitalization (days)**		**Mortality**		**Nonfatal CBV**	
	**NAC**	**Control**	**NAC**	**Control**	**NAC**	**Control**	**NAC**	**Control**	**NAC**	**Control**
Ozaydin 2008 [12]	3/58	12/57	NA	NA	7.7 ± 3	7.9 ± 4.2	0/58	2/57	1/58	0/57
Kazemi 2013 [16]	14/120	19/120	120 ± 45.6	115.2 ± 79.2	7.4 ± 1.3	7.2 ± 0.9	1/120	2/120	1/120	1/120
Eren 2003 [19]	2/10	1/10	NA	NA	NA	NA	0/10	0/10	0/10	0/10
Orhan 2006 [20]	0/10	1/10	23.2 ± 1.75	22.6 ± 1.84	7.2 ± 0.42	7.3 ± 0.48	0/10	0/10	NA	NA
Ozaydin 2013 [21]	9/104	25/104	NA	NA	NA	NA	1/104	2/104	2/104	0/104
Peker 2008 [22]	0/19	2/21	NA	NA	NA	NA	0/19	0/21	0/19	0/21
Wijeysundera 2007 [23]	50/88	58/87	45.6	40.8*	8 (6–12)	8 (6–12)†	0/88	7/87	4/88	4/87
EI-Hamamsy 2007 [24]	4/50	6/50	NA	NA	5.4 ± 2.3	5.3 ± 2.5	3/50	0/50	0/50	0/50
Haase 2007 [25]	19/30	16/30	44	45*	8(7–11)	8(7–11)†	0/30	1/30	NA	NA
Kim 2011 [26]	4/24	8/24	72 ± 36	81.6 ± 50.4	11.3 ± 6.3	10.5 ± 4.5	0/24	2/24	0/24	0/24

Data are number or mean ± deviation; CBV, cerebrovascular events; NA, data not available; *values expressed as median; †data expressed as median (interquartile range).

**Table 3:** POAF outcome definition and assessment

**Reference**	**Method of atrial fibrillation assessment**	**Definition of atrial fibrillation**
Ozaydin 2008 [12]	ECGs performed continuously at the first 2 postoperative days in the ICU, and 2 times a day routinely when new symptom developed or observed in the wards.	An irregular narrow complex rhythm with absence of discrete p-waves lasting longer than 5 minutes
Kazemi 2013 [16]	Holter performed continuously for 72 h after surgery	More than 5 minutes of AF or associated with hemodynamic compromise requiring therapy immediately.
Eren 2003 [19]	ECGs were recorded on the first postoperative day	Not reported
Orhan 2006 [20]	Not reported	Not reported
Ozaydin 2013 [21]	ECGs performed continuously during ICU stay and all-day Holter was used during the rest of hospitalization.	The incidence of AF lasting longer than 5 minutes during hospitalization
Peker 2008 [22]	ECGs conducted continuously during the first 2 postoperative days in the ICU, and 2 times per day routinely when new symptom developed or noted	Not reported
Wijeysundera 2007 [23]	Continuous telemetry or 12-lead ECGs	Any new atrial fibrillation
EI-Hamamsy 2007 [24]	Not reported	Not reported
Haase 2007 [25]	Not reported	Not reported
Kim 2011 [26]	Not reported	Not reported

ECG, electrocardiogram.

**Figure 2:**
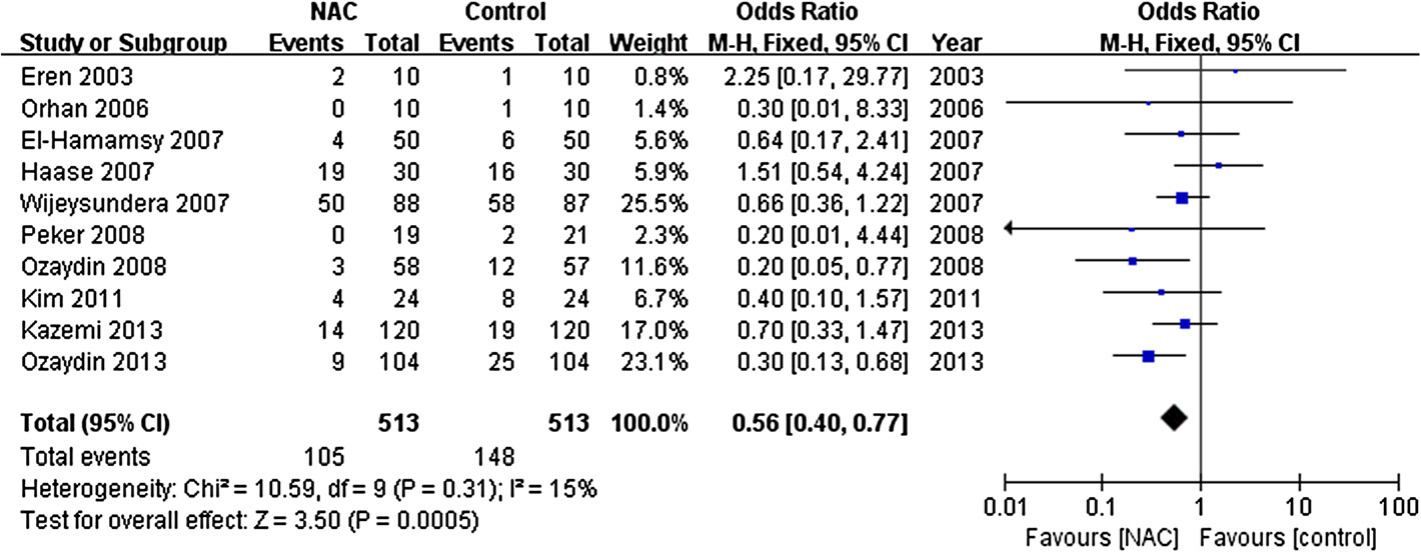
Effects of N-acetylcysteine on the prevention of postoperative atrial fibrillation.

Pooling all ten RCTs, 20.5% (105 of 513) of patients given NAC and 28.8% (148/513) of controls developed POAF. The meta-analysis of ten trials using a fixed-effects model showed that NAC reduced the incidence of POAF (OR 0.56, 95% CI 0.40 to 0.77; *P* < 0.001; Figure 2) compared with controls, with no heterogeneity between the studies (I^2^ = 15%, *P* = 0.31).

**Figure 3:**
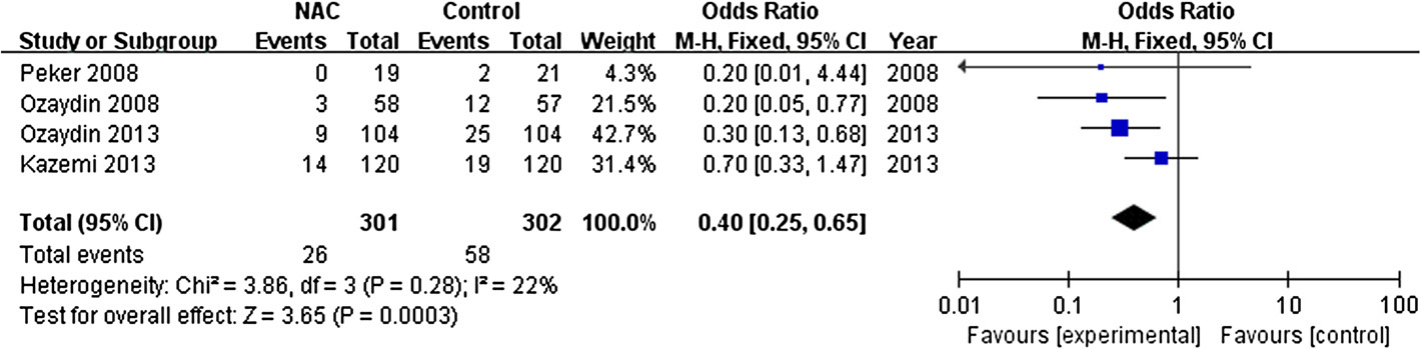
Effects of long-term N-acetylcysteine administration on the prevention of postoperative atrial fibrillation.

**Figure 4:**
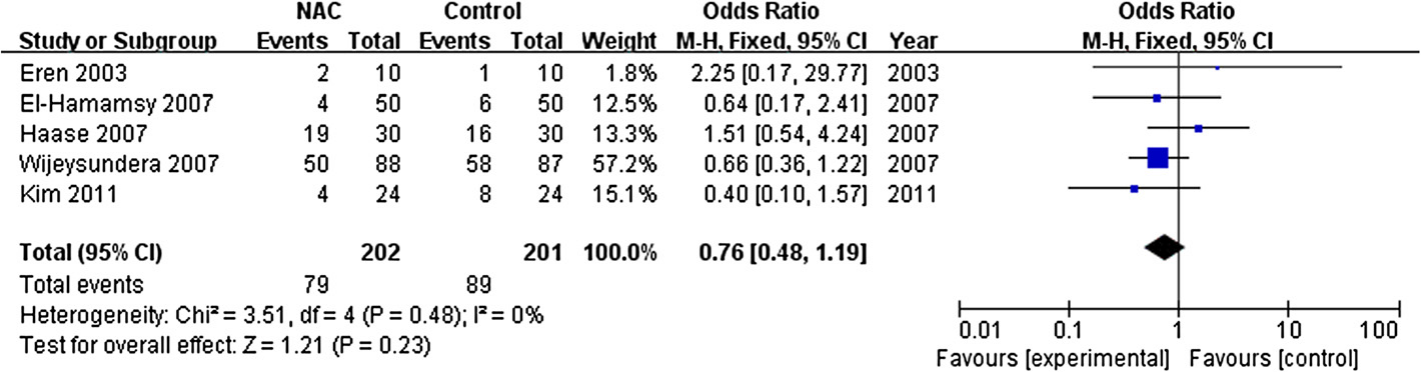
Effects of short-term N-acetylcysteine administration on the prevention of postoperative atrial fibrillation.

Sensitivity analyses were conducted to test the robustness of the main analysis. We tested whether excluding small sample studies (n <60 patients) [19,20,22,26] would change the direction of the overall result. The meta-analysis of the remaining studies (OR 0.57, 95% CI 0.40 to 0.80; *P* = 0.001) [12,16,21,23-25] was similar to the overall result, with little statistical heterogeneity (I^2^ = 42%, *P* = 0.12). A trial [25] enrolled some patients with a history of AF, but the pooled results (OR 0.50; 95% CI 0.35 to 0.71; *P* < 0.001; I^2^ = 0%, *P*_heterogeneity_ = 0.56) did not change when this trial was exclude. In addition, we tested whether different duration of NAC supplementation after cardiac surgery would alter the direction of the overall result. Using the fixed-effects model, the meta-analysis of studies (OR 0.40; 95% CI 0.25 to 0.65; *P* < 0.001; I^2^ = 22%, *P*_heterogeneity_ =0.28; Figure 3) [12,16,21,22] with a long-term (lasting for 48 h-72 h after operation) was similar to the overall effects (OR 0.56, 95% CI 0.40 to 0.77). However, it failed to reduce the incidence of POAF with a short-term NAC (within 24 h after surgery) supplementation (OR 0.64; 95% CI 0.39 to 1.06; *P* = 0.09; I^2^ = 0%, *P*_heterogeneity_ =0.7; Figure 4) [19,23-26].

### Length of ICU stay

Five studies [16,20,23,25,26] reported the data of ICU length of stay, while only three [16,20,26] studies reported the values as mean ± standard deviation. All the data were converted from days into hours for analysis. Prophylactic NAC was not associated with a reduction in ICU length of stay (WMD 0.60; 95% CI −0.97 to 2.16; *P* = 0.45). No heterogeneity of included studies was noted (I^2^ = 0%, *P* = 0.64).

### Hospital length of stay

**Figure 5:**
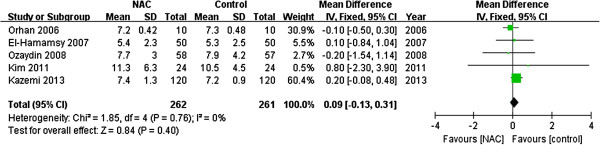
Effects of N-acetylcysteine on the length of hospital stay.

Five [12,16,20,24,26] studies reported values as mean ± standard deviation, two [23,25] expressed data as median and interquartile range, and one [21] showed values by bar graph. The meta-analysis of the five studies using a fixed-effects model presented that NAC treatment did not reduce the length of hospital stay (WMD 0.09; 95% CI −0.13 to 0.31; *P* = 0.40; Figure 5). No statistical heterogeneity was observed across studies (I^2^ = 0%, *P* = 0.76).

### All-cause mortality

**Figure 6:**
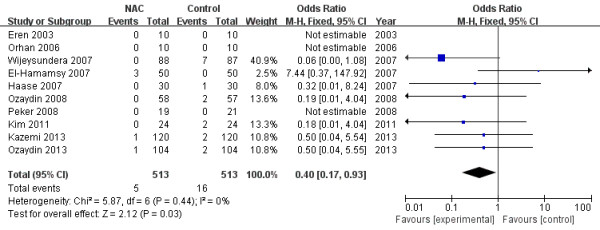
Effects of N-acetylcysteine on the all-cause mortality.

Death occurred in 5 of 513 patients (0.9%) treated with NAC and in 16 of 513 patients (3.1%) treated with placebo. Use of NAC was associated with a reduction in all-cause death (OR 0.40; 95% CI 0.17 to 0.93; *P* = 0.03; I^2^ = 0%, *P*_heterogeneity_ =0.44; Figure 6).

### Incidence of cerebrovascular events

Seven [12,16,19,21-23,26] studies reported the incidence of cerebrovascular events after operation. Cerebrovascular events occurred in 8 of 423 patients (1.9%) treated with NAC and in 5 of 424 patients (1.2%) treated with placebo. Prophylactic use of NAC failed to present a reduction in cerebrovascular accidents (OR 1.68; 95% CI 0.60 to 4.69; *P* = 0.32; I^2^ = 0%, *P*_heterogeneity_ =0.64).

### Publication bias

**Figure 7:**
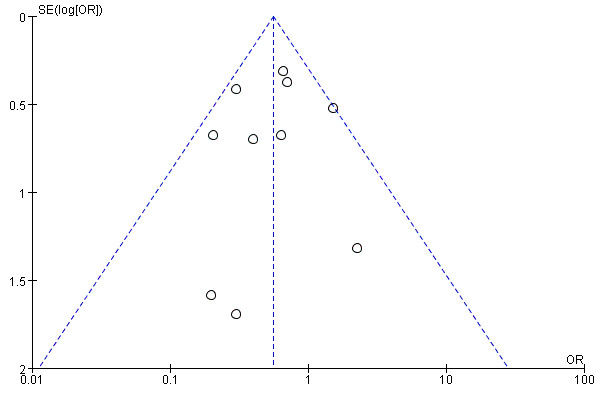
Funnel plot of N-acetylcysteine on the prevention of atrial fibrillation after cardiac surgery.

The funnel plot for the incidence of POAF was shown in Figure 7. The funnel plot appeared symmetrical, suggesting that there was no potential publication bias among the included trials.

## Discussion

This meta-analysis shows that prophylactic NAC can reduce the incidence of POAF in adult patients undergoing cardiac surgery, with a conclusion similar to the previous meta-analyses [14,15]. However, the meta-analysis of trials (OR 0.64; 95% CI 0.39 to 1.06) using a short-term NAC administration, differing from the pooled effect of studies with a long-term (OR 0.51; 95% CI 0.33 to 0.79), demonstrates that there is no significant difference compared with controls. These findings suggest that NAC can be used for adult patients undergoing cardiac surgery to prevent POAF. It would be more reasonable to prolong the duration of NAC administration up to postoperative day 2–3. Previous studies presented that on postoperative day 2–3 the inflammatory cytokines levels are the highest, corresponding to the day of the highest incidence of POAF [7,27]. To some extent, the different results between short-term and long-term NAC administration may be associated with the anti-inflammatory property of NAC. Additionally, NAC has a generally good safety profile [16,21,23]. Of the 10 included studies, only one [23] reported evident side effects among patients, but there was no statistical difference when compared with control group.

An increasing body of evidence demonstrates that oxidative stress and inflammatory reaction play an important role in the pathophysiology of POAF [7,9,28]. Antioxidants, including NAC, have proved to decrease serum levels of molecules markers of cellular oxidative stress in patients undergoing heart surgery [9-11,16,28,29]. NAC is a glutathione precursor, by entering cells and being hydrolyzed to cysteine, it stimulates glutathione synthesis [12,16]. In this way, it increases the level of intracellular reduced glutathione, which is often depleted as a response to increased status of inflammation reaction and oxidative stress [16,30]. In addition to that, it may also block renin angiotensin system and/or atrial remodeling via its antioxidant actions and anti-inflammatory [12,20,31]. Thus, NAC is a potential agent used for reducing the incidence of POAF in adult patients undergoing cardiac surgery.

Although POAF is often considered both transient and self-limiting [7,15], it can increase the length of ICU and hospital stay, cerebrovascular events, and mortality [3-7]. With regard to ICU and hospital stay, the pooled effects are similar to the pre-existing meta-analyses [13-15], showing no significant difference compared with controls. There is also no significant reduction in the risk of cerebrovascular events (OR 1.68; 95% CI 0.60 to 4.69) between two groups. This meta-analysis, however, demonstrates that prophylactic NAC apparently reduce the occurrence of all-cause mortality (OR 0.40; 95% CI 0.17 to 0.93), which differs from the previous meta-analysis (OR 0.81; 95% CI 0.39 to 1.68) conducted by Wang et al. [13].

There are several limitations to this meta-analysis that should be noted. First, there is significant heterogeneity in both the methods of monitoring and the definition of POAF between the studies. AF following cardiac surgery was reported as a clinical outcome in seven studies [19,20,22-26], and only three studies [12,16,21] treated POAF as a primary endpoint. All these may lead to potential overestimation or underestimation of the true incidence of POAF. Second, for the POAF endpoint, the use of beta-blockers and amiodarone was not the standard therapy among most included studies [12,16,19,20,22-26]. Meanwhile, the adjunctive use of NAC in addition to these proven prophylactic strategies is not known. An adjunctive prophylactic protocol, however, that might further prevent POAF without reducing blood prssure and/or heart rate. As such, Ozaydin and his colleagues demonstrated that carvedilol plus NAC significantly reduced the incidence of POAF compared with carvedilol plus placebo [21]. Next, due to different NAC doses and schedules used in these trials, we are unable to evaluate this heterogeneity on clinical outcomes and identify an optimal NAC dose from this meta-analysis. Finally, the positive effect seen is based almost entirely on two trials from the same group [12,21], however, the results of which have not been re-produced in the majority of the other trials.

Future studies should aim to treat the incidence of POAF as a primary endpoint, and standardize the definition of POAF. In addition, these studies should compare various does of NAC, including varying lengths of therapy. It is also important to assess the impact of NAC supplementation on the top of already proven agents for preventing POAF, including beta-blockers, amiodarone, and any other potential prophylaxis.

## Conclusions

This meta-analysis shows that prophylactic use of NAC could reduce the incidence of POAF and all-cause mortality for adult patients undergoing cardiac surgery. However, larger RCTs evaluating these and other postoperative complication endpoints are needed.

## Competing interests

The authors declare that they have no competing interests.

## Authors’ contributions

LXH conceived the study, participated in the design, collected the data, performed statistical analyses and drafted the manuscript. XCY helped to collect data. FGH conceived the study, participated the design. All authors read and approved the final manuscript.

## Pre-publication history

The pre-publication history for this paper can be accessed here:

http://www.biomedcentral.com/1471-2261/14/52/prepub
